# Physiological, Cytological and Transcriptome Analysis of a Yellow–Green Leaf Mutant in *Magnolia sinostellata*

**DOI:** 10.3390/plants14071037

**Published:** 2025-03-27

**Authors:** Xiawen Zhou, Shaozong Yang, Fangwei Zhou, Liang Xu, Congguang Shi, Qiuling He

**Affiliations:** 1 Zhejiang Academy of Forestry, No.399, Liuhe Road, Hangzhou 310023, China; xiaxia7051129@163.com (X.Z.); yangsz863@163.com (S.Y.); zhoufangwei@njfu.edu.cn (F.Z.); jachary@163.com (L.X.); 2 Zhejiang Province Key Laboratory of Plant Secondary Metabolism and Regulation, College of Life Sciences and Medicine, Zhejiang Sci-Tech University, Hangzhou 310018, China

**Keywords:** *Magnolia sinostellata*, physiology, cytology, transcriptomics, chlorophyll, chloroplast

## Abstract

Leaf color mutants serve as excellent models for investigating the metabolic pathways involved in chlorophyll biosynthesis, chloroplast development, and photosynthesis in plants. This study aimed to elucidate the mechanisms underlying color formation in the yellow–green leaf mutant (YL) of *Magnolia sinostellata* by employing physiological, cytological and transcriptomic analyses to compare the mutant with control plants (wild type *Magnolia sinostellata*, WT). Physiological assessments revealed a reduction in chlorophyll content, particularly chlorophyll b, alongside an increase in the flavonoid level in YL relative to WT. Cytological examinations indicated the presence of defective chloroplasts within the mesophyll cells of the mutants. Transcriptomic analysis identified 8205 differentially expressed genes, with 4159 upregulated and 4046 downregulated. Genes associated with chlorophyll metabolism, flavonoid metabolism, photosynthesis, and signaling pathways were found to play crucial roles in leaf yellowing. In conclusion, this study delineated the phenotypic, physiological, cytological, and transcriptomic differences between YL and WT leaves, offering novel insights into the mechanisms driving leaf yellowing in *Magnolia sinostellata*.

## 1. Introduction

Leaf color variation is a prevalent phenomenon among plants, with different fertility stages often exhibiting distinct leaf colors [1]. Leaf color mutants serve as valuable resources for investigating pigment metabolism, chloroplast development and differentiation, photosynthesis, and other related pathways, while also offering critical insights for cultivar enhancement [2]. Plant leaves contain a diverse array of pigments, including chlorophyll, lutein, anthocyanins, and carotenoids. Alterations in the coloration of mutant leaves are typically linked to shifts in the relative proportions of these pigments [3]. For example, during the coloration phase, the chlorophyll content in the leaves of maple trees (*Liquidambar formosana* Hance) decreases, while the anthocyanin content increases significantly [4].

Chlorophyll synthesis takes place within chloroplasts, with the structural integrity of chloroplasts serving as a fundamental basis for this process [5]. The yellow–green leaf mutant of Hami melon (*Cucumis melo* L.) exhibits impaired chloroplast development characterized by a significant reduction in the number and organization of thylakoid grana lamellae [6]. Numerous studies have indicated that genetic factors play a crucial role in leaf color mutations, often linked to mechanisms of chlorophyll synthesis and degradation. In *Populus deltoides* marsh yellow leaf mutants, there is a downregulation of genes involved in chlorophyll biosynthesis and an upregulation of chlorophyllase genes associated with chlorophyll degradation [7]. It has been demonstrated that mutations in genes encoding enzymes such as PBGD [8], MgPCY [9], POR [10], and DVR [11] result in a loss of enzyme function, thereby obstructing chlorophyll synthesis and leading to varying degrees of green loss in plant leaves.

*Magnolia sinostellata*, an endangered deciduous shrub within the genus *Magnolia*, is noted for its significant ornamental value [12]. *Magnolia sinostellata* is an ornamental plant, with a peculiar flower shape and varied flower color, which is highly ornamental, but the flowering period of *Magnolia sinostellata* is only about two weeks, which is a short ornamental period [13]. The cultivation direction of *Magnolia sinostellata* varieties is mainly focused on the flower color, flower type and other aspects, with less focus on the study of leaf color [14]. The molecular mechanisms underlying its leaf color variation remain inadequately understood. A naturally mutated *Magnolia sinostellata* yellow–green leaf mutant strain was found in the breeding base of *Magnolia sinostellata* in Sidu township, Lishui city, Zhejiang province, and its traits were stabilized after grafting and propagation. 

In this study, we investigated the physiological, cytological and molecular differences between the yellow–green leaf mutant (YL) and wild-type (WT) specimens. Our objective was to elucidate the pigment and chloroplast structural modifications contributing to leaf yellowing and to identify key genes associated with leaf color changes through transcriptome sequencing and reverse transcriptase real-time quantitative polymerase chain reaction (qRT–PCR) analysis. Exploring the mechanism of the leaf color mutation in *Magnolia sinostellata* can provide a certain theoretical basis for enhancing the ornamental properties of *Magnolia sinostellata* in the leaf spreading period, lengthening the ornamental period of *Magnolia sinostellata*, and cultivating new varieties of *Magnolia sinostellata* with attractive foliage.

## 2. Results

### 2.1. Pigment Contents of Leaves

**Figure 1:**
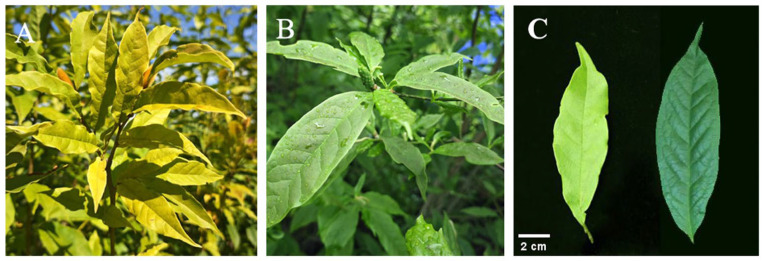
The phenotypic characteristics of yellow–green leaf mutants (YL) and wild-type (WT) plants of *Magnolia sinostellata*. (**A**) YL plants; (**B**) WT plants; (**C**) leaf of YL (left) and WT (right). Scale bars: 2 cm (**C**).

**Figure 2:**
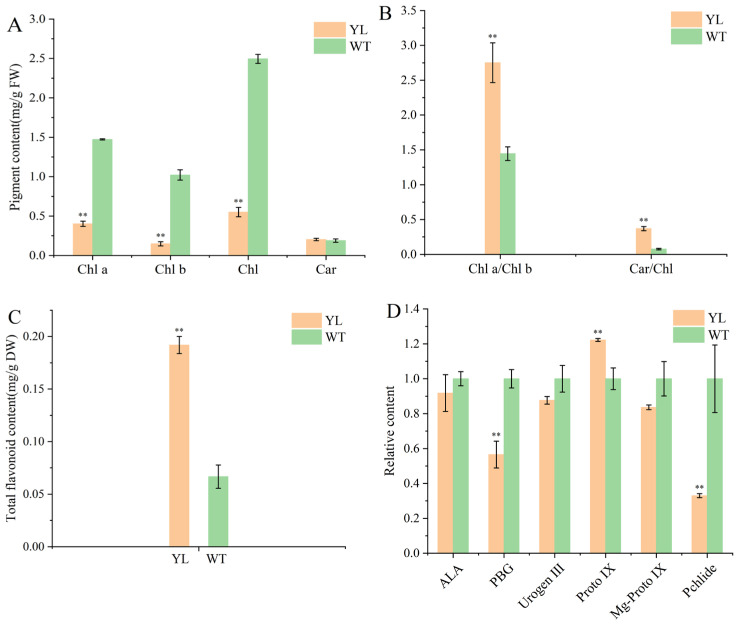
Pigment contents in leaves of yellow–green leaf mutants (YL) and wild-type (WT) leaves of *Magnolia sinostellata*. (**A**) Chlorophyll (Chl) and carotenoid (Car) content in mg/g fresh weight; (**B**) the ratio of Chl a to Chl b and Car to Chl; (**C**) total content of flavonoids in YL and WT leaves. (**D**) Analysis of relative chlorophyll intermediary contents in YL and WT leaves. Student’s *t*-test was used to identify significant differences between mutant and green leaves (** *p* < 0.01). ALA, 5-aminolevulinic acid; PBG, porphobilinogen; Urogen III, uroporphyrinogen III; Proto Ⅸ, protoporphyrin IX; Mg-Proto IX, Mg-protoporphyrin IX; Pchlide, protochlorophyllide.

The mutant exhibited a spontaneous mutation characterized by a yellow–green leaf phenotype (Figure 1). To examine alterations in chlorophyll content within the mutant, we analyzed the levels of key pigments in both the mutant and wild-type (WT) plants. The chlorophyll a, chlorophyll b, total chlorophyll, and carotenoid contents in the mutant were measured at 0.40 mg/g, 0.15 mg/g, 0.55 mg/g, and 0.20 mg/g, respectively. Mutant leaves displayed a significant reduction in chlorophyll a, chlorophyll b, and total chlorophyll contents compared to WT, while carotenoid levels remained relatively unchanged (Figure 2A). The reduction in chlorophyll b was more pronounced than that of chlorophyll a, resulting in an elevated chlorophyll a/b ratio in the mutant leaves. Despite the absence of a significant difference in carotenoid content between mutant and WT leaves, the Car/Chl ratio was increased in the mutant due to the diminished chlorophyll content (Figure 2B). Furthermore, the total flavonoid content in the yellow–green leaves (YL) was significantly higher than that in WT leaves (Figure 2C). These findings suggest that the yellow leaf phenotype of the mutant is associated with decreased chlorophyll content, increased total flavonoid content, and alterations in the pigment ratios of Car/Chl and Chl a/Chl b.

Furthermore, we conducted a comparative analysis of the precursor content involved in chlorophyll synthesis between the mutant and wild-type (WT) plants. Compared with WT, porphobilinogen (PBG) was significantly reduced in YL leaves, followed by a prominent increase in protoporphyrin IX (Proto IX). However, the content of protochlorophyllide (Pchlide), which is located downstream of the chlorophyll synthesis pathway, was markedly reduced. It was inferred that chlorophyll biosynthesis in YL leaves might be blocked between Proto IX and Pchlide, suggesting that the decrease in chlorophyll content in YL leaves was closely related to blockage of the chlorophyll biosynthesis pathway (Figure 2D).

### 2.2. Cytological Observations

**Figure 3:**
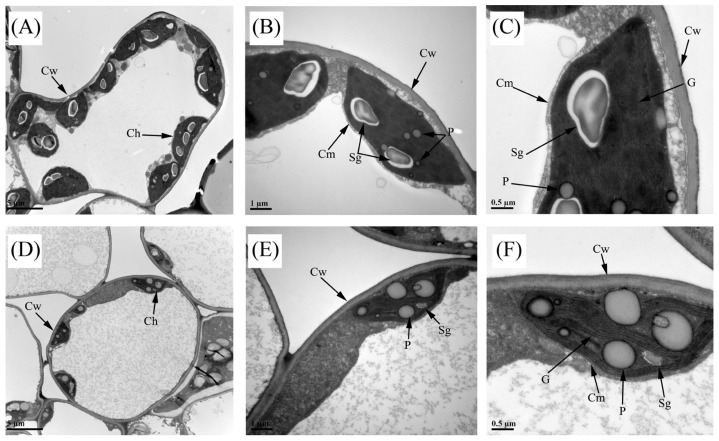
Transmission electron microscopic images of chloroplasts in yellow–green leaf mutants (YL) and wild-type (WT) leaves of *Magnolia sinostellata*. (**A**–**C**) Chloroplast ultrastructure in WT; (**D**–**F**) chloroplast ultrastructure in YL. Cw, cell wall; Ch, chloroplast; Cm, chloroplast membrane; Sg, starch grain; G, grana layer; P, plastoglobule. Scale bars: 5 μm (**A**,**D**), 1 μm (**B**,**E**), 0.5 μm (**C**,**F**).

To further investigate the relationship between chloroplast structure and changes in leaf coloration, we performed ultrastructural examinations of WT and YL leaves. In the WT, chloroplasts exhibited an intact double-membrane structure, and the interior of the chloroplasts contained many flattened thylakoids derived from the inner membrane, as well as grana stacked by the thylakoids. In contrast, YL chloroplasts displayed a blurred bilayer membrane structure and sparse layers of stroma lamella. In addition, most of the WT chloroplasts contained one or two large starch grains and a few smaller plastoglobules, whereas in YL chloroplasts almost no starch grains were observed but many larger plastoglobules were observed (Figure 3). As previously outlined, chloroplasts in the mutant leaves exhibited structural abnormalities that potentially impeded their normal biological functions, resulting in a yellowish–green appearance of the leaves.

### 2.3. Transcriptomics Analysis

**Figure 4:**
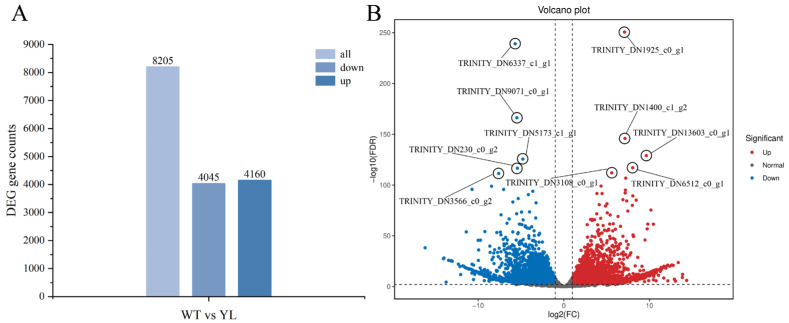
Identified differentially expressed genes between WT and YL leaves. (**A**) DEG counts in YL compared with WT. (**B**) Volcano plot of all DEGs in YL compared with WT.

To investigate the molecular mechanisms underlying the yellow–green leaf phenotype in the mutant, we conducted high-throughput sequencing of leaf construct libraries from both mutant and wild-type specimens. Following the removal of low-quality sequences, filtering of artifacts, and exclusion of ambiguous reads, we obtained a total of 43.87 Gb of clean bases. Differential gene expression analysis was subsequently performed between the wild-type (WT) and yellow–green leaf (YL) samples. This analysis identified 8205 differentially expressed genes (DEGs) with a fold change of ≥2 and a false discovery rate (FDR) of <0.01, comprising 4160 upregulated and 4045 downregulated genes (Figure 4).

**Figure 5:**
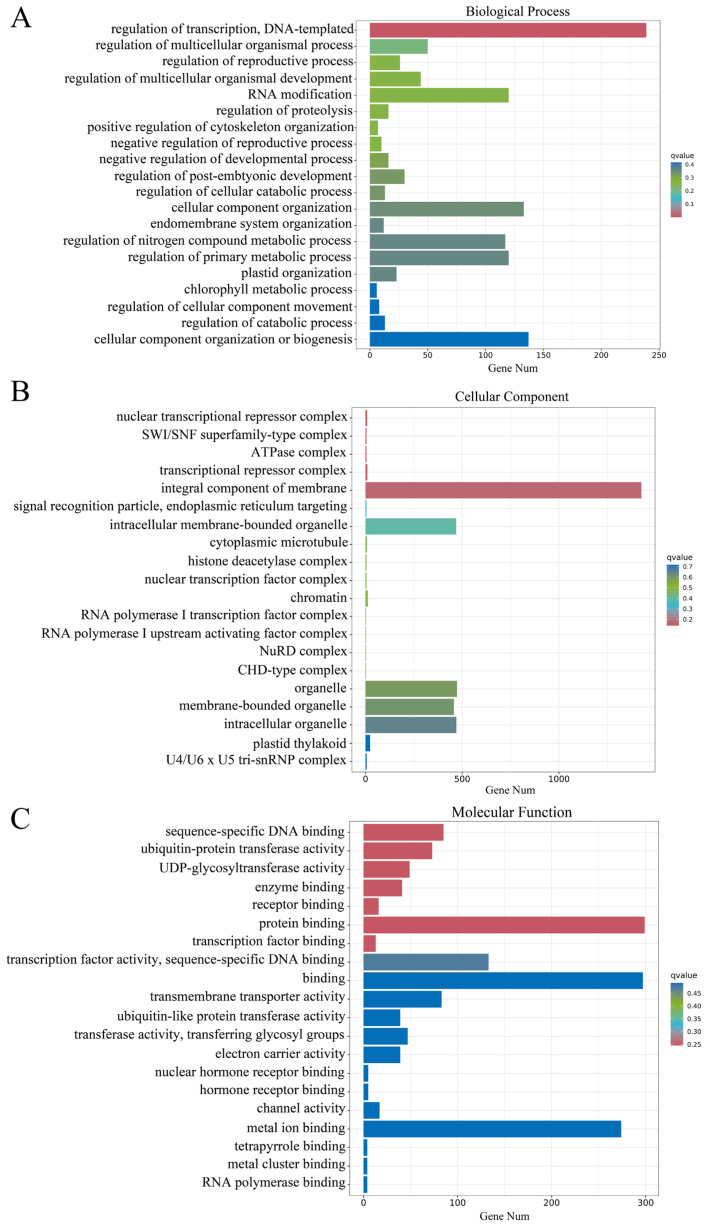
Differentially expressed genes (DEGs) analysis based on Gene ontology (GO). (**A**) DEGs in biological process; (**B**) DEGs in cellular component; (**C**) DEGs in molecular function.

GO enrichment analysis classified the DEGs into three categories: biological process, cellular component and molecular function. In biological process, the DEGs were mainly enriched in the regulation of transcription, DNA templates, cellular component organization or biogenesis, plastid organization and chlorophyll metabolism process (Figure 5A); in cellular components, the DEGs were mainly enriched in binding, transcription factor activity, and protein binding (Figure 5B); in molecular function, the DEGs were mainly enriched in the integral component of membrane, intracellular organelle, and plastid thylakoid (Figure 5C). Among them, the differential expression of genes in chlorophyll metabolism processes may be responsible for the reduced chlorophyll content in YL. The differential expression of genes in plastid organization and plastid thylakoid may be closely related to the abnormal chloroplast structure in YL. Other differentially expressed genes were enriched in transcription factor activities, indicating that transcriptional regulation was active in YL, and that YL may differentially express genes related to leaf color variation mainly through regulation at the transcriptional level to regulate leaf color changes.

**Figure 6:**
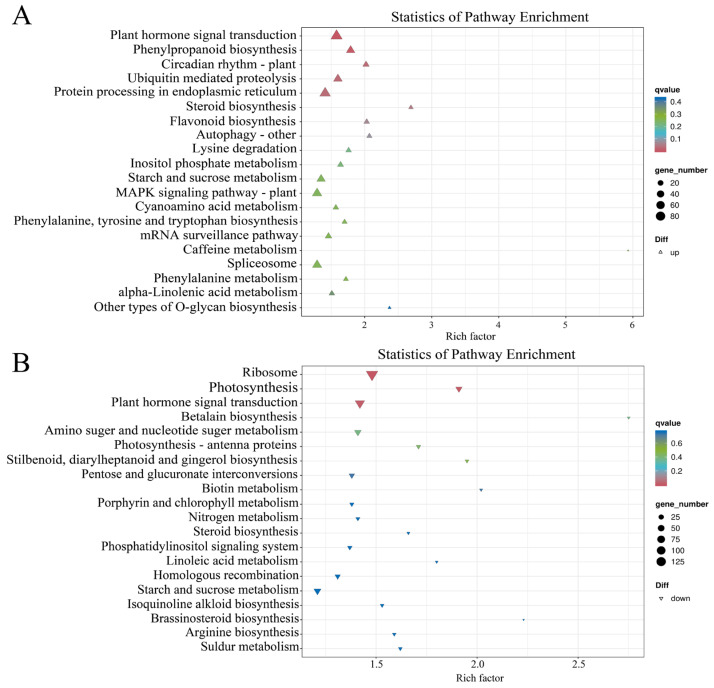
Differentially expressed genes (DEGs) analysis based on Kyoto Encyclopedia of Genes and Genomes (KEGG) pathways. (**A**) Upregulated DEGs; (**B**) Downregulated DEGs.

In addition, KEGG enrichment analysis showed that the up-regulated DEGs were significantly enriched in the pathways of plant hormone signaling transduction, phenylpropanoid biosynthesis, flavonoid biosynthesis, and MAPK signaling pathway-plant (Figure 6A). Down-regulated DEGs were notably enriched in the pathways of photosynthesis, photosynthesis antenna proteins, and porphyrin and chlorophyll metabolism (Figure 6B). These data suggest that the differential expression of the related genes in the porphyrin and chlorophyll metabolism pathways, photosynthesis, and flavonoid biosynthesis pathway may be closely related to the changes in YL leaf pigmentation. In addition, the plant hormone signaling transduction and MAPK signaling pathway-plant pathway were enriched and striking, showing an important role in regulating leaf color variation in *Magnolia sinostellata*.

#### 2.3.1. Differentially Expressed Genes Involved in Chlorophyll Anabolism and Photosynthesis

**Figure 7:**
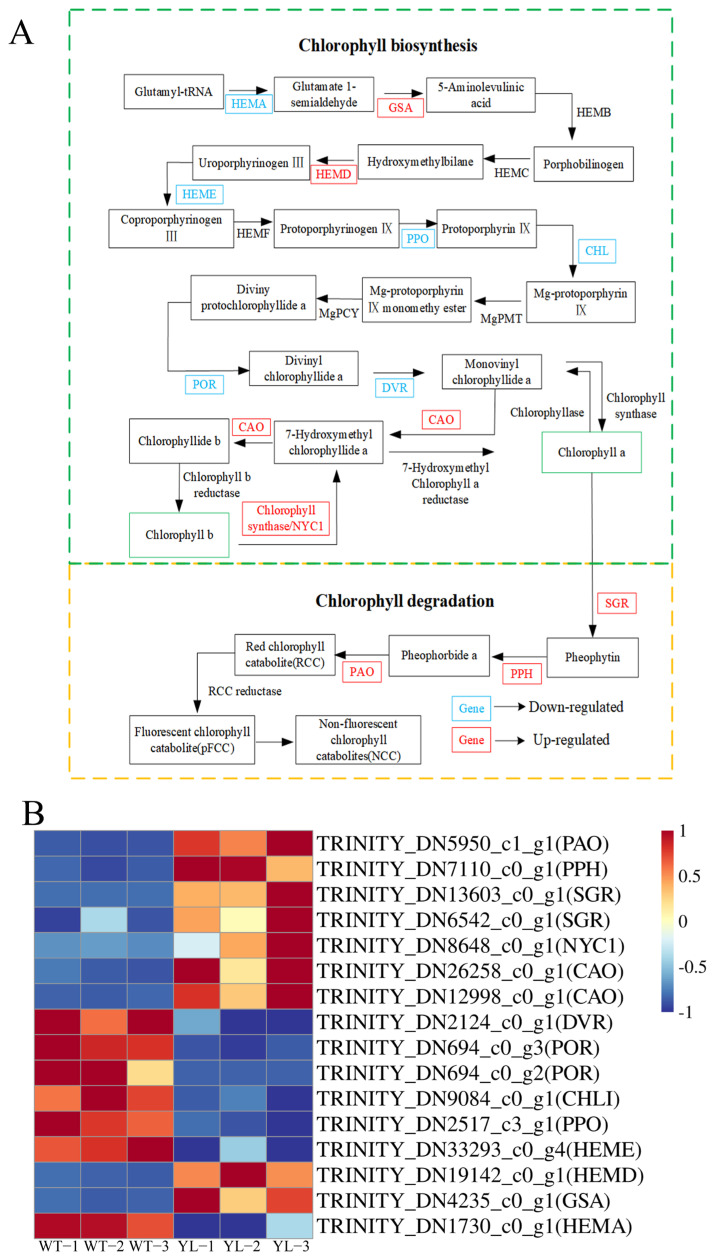
Expression profiles of differently expressed genes (DEGs) between WT and YL involved in chlorophyll metabolism. (**A**) Chlorophyll biosynthesis and metabolic pathways. (**B**) Expression of DEGs involved in chlorophyll biosynthesis and metabolic pathways. The expression level was calculated from three biological replications scaled by DESeq2. The color bar indicates an increasing expression level from blue to red.

The chlorophyll anabolic pathway plays a crucial role in leaf yellowing. A total of 16 differentially expressed genes encoding 13 enzymes were identified as being associated with chlorophyll anabolism. In the yellow leaf mutant, six genes involved in chlorophyll synthesis—specifically encoding *HEMA*, *HEME*, *PPO*, *CHL*, *POR*, and *DVR*—were significantly downregulated. Conversely, the expression of several genes associated with chlorophyll degradation, including *PPH*, *PAO*, *NYC1*, and *SGR*, was upregulated (Figure 7). These alterations in gene expression are intimately linked to the processes of chlorophyll synthesis and degradation, leading to a reduction in leaf chlorophyll content and a subsequent change in leaf coloration.

**Figure 8:**
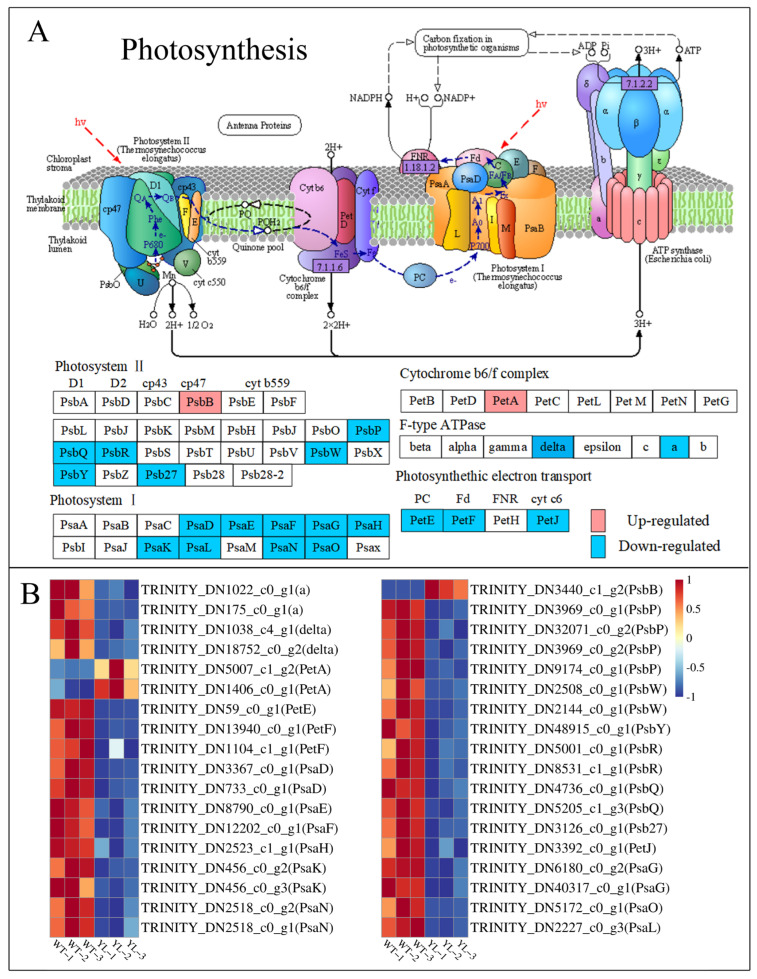
Expression profiles of differently expressed genes (DEGs) between WT and YL involved in photosynthesis. (**A**) Schematic illustrations of photosynthesis; (**B**) expression of DEGs involved in photosynthesis. The expression level was calculated from three biological replications and scaled using DESeq2. The color bar indicates an increasing expression level from blue to red. Schematic illustrations were modified from the KEGG website (https://www.kegg.jp/ (accessed on 22 August 2024)).

**Figure 9:**
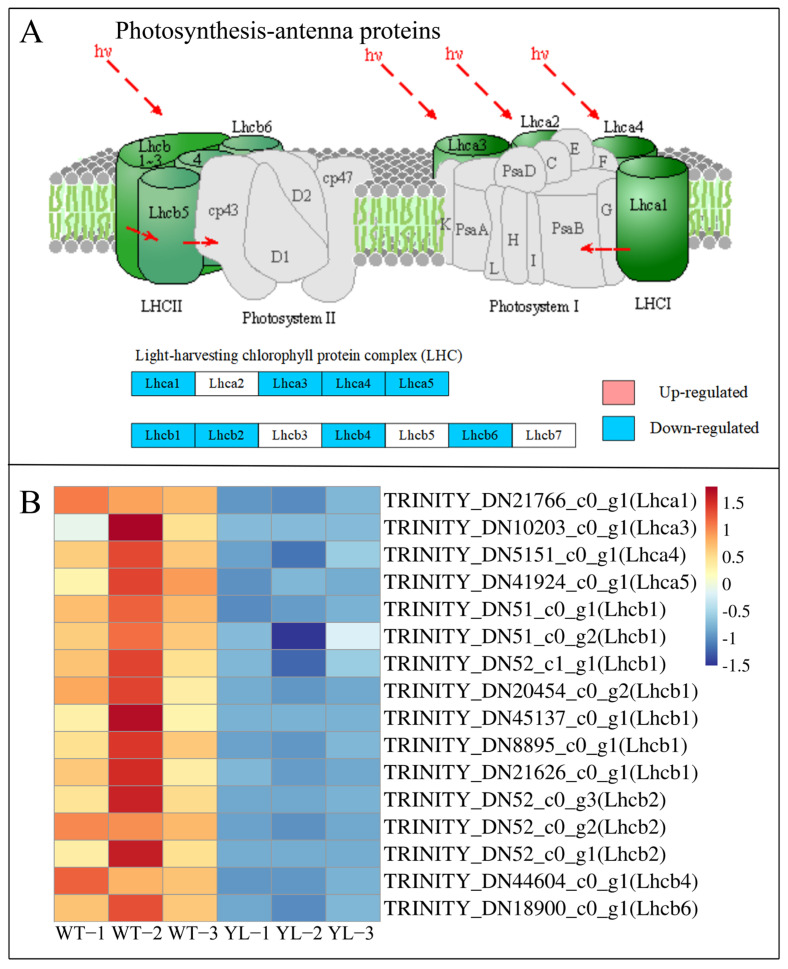
Expression profiles of differently expressed genes (DEGs) between WT and YL involved in photosynthesis antenna proteins. (**A**) Schematic illustrations of photosynthesis antenna proteins; (**B**) Expression of DEGs involved in photosynthesis antenna proteins. The expression level was calculated from three biological replications and scaled using DESeq2. The color bar indicates an increasing expression level from blue to red. Schematic illustrations were modified from the KEGG website (https://www.kegg.jp/ (accessed on 22 August 2024)).

The function and structure of chloroplasts are intricately connected to photosynthesis, and as many as 50 differentially expressed genes (DEGs) related to photosynthesis and photosynthesis antenna proteins exhibited pronounced downregulation in the yellow leaf mutant (Figure 8 and Figure 9). This suggests that the normal functioning of chloroplasts in the yellow leaf mutant may be compromised, resulting in diminished photosynthetic efficiency.

Although there is some variability between replicates due to biological samples or experimental manipulation, the trend of up- or down-regulation of genes is consistent, and it can be judged from these data that chlorophyll synthesis and photosynthesis are inhibited in YL.

#### 2.3.2. Differentially Expressed Genes Involved in Flavonoid Biosynthesis

**Figure 10:**
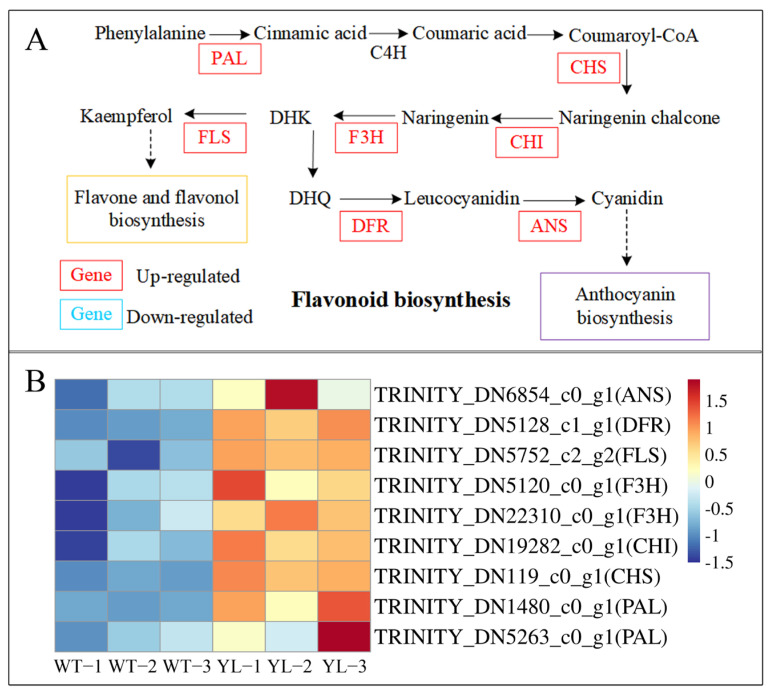
Expression profiles of differently expressed genes (DEGs) between WT and YL involved in flavonoid biosynthesis. (**A**) Flavonoid biosynthesis pathway; (**B**) expression of DEGs involved in flavonoid biosynthesis pathway. The expression level was calculated from three biological replications scaled by DESeq2. The color bar indicates an increasing expression level from blue to red.

Flavonoids, predominantly yellow or yellowish pigments, are extensively distributed in plants and constitute essential components of phytochromes. KEGG analysis identified nine genes with upregulated expression that encode key enzymes within the flavonoid biosynthesis pathway (Figure 10). Notably, the expression of several upstream enzyme genes, including *PAL*, *CHS* and *CHI*, was enhanced, potentially leading to a comprehensive upregulation of the flavonoid biosynthesis pathway and an increase in total flavonoid production.

### 2.4. Validation of Gene Expression by qRT–PCR

**Figure 11:**
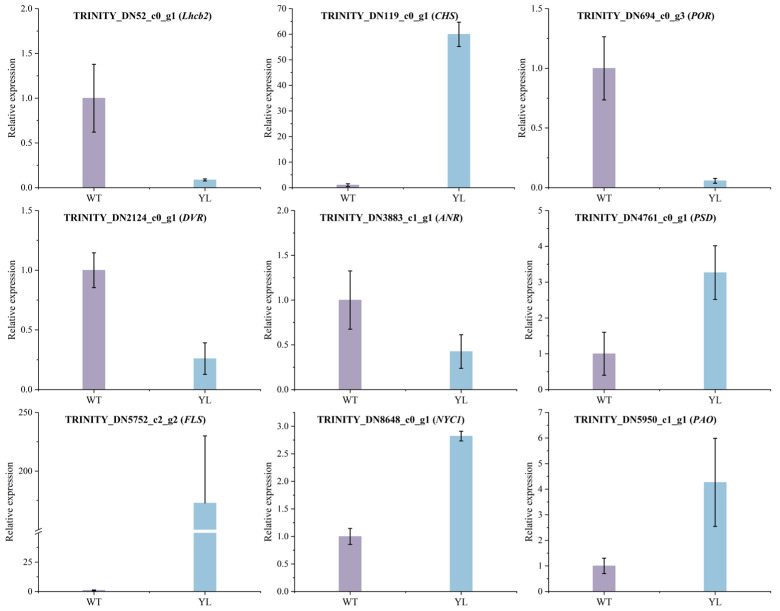
qRT–PCR verification of the expression levels of 9 DEGs identified by RNA–seq.

**Figure 12:**
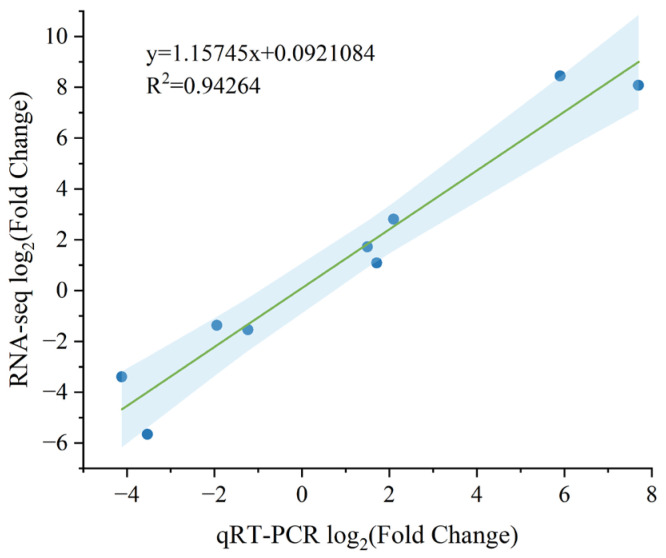
Correlation analysis of qRT–PCR and RNA–seq data. The bule shadow means 95% confidence interval.

To validate the gene predictions derived from transcriptomic analysis, nine differentially expressed genes (DEGs) were selected for quantitative real-time RT–PCR (qRT-PCR) analysis (Figure 11). The expression patterns observed were generally consistent with the transcriptomic data. Compared with the WT, the relative expression levels of *DVR* and *POR*, which are involved in chlorophyll synthesis, were lower in YL, while the relative expression levels of *PAO* and *NYC1*, involved in chlorophyll degradation, were upregulated. In the flavonoid synthesis pathway, the levels of *CHS*, *FLS* and *Lhcb2*, involved in photosynthesis, were different between YL and WT. Correlation analysis (Figure 12) showed that there was a positive correlation between the results of qRT–PCR and the transcriptome data (R^2^ = 0.94264), which proved the reliability of the transcriptome data.

## 3. Discussion

### 3.1. Leaf Yellowing Due to Changes in Leaf Pigmentation Ratios

Chlorophyll serves as the primary pigment influencing leaf coloration, with variations in leaf color typically attributed to chlorophyll degradation [15]. Differences in the relative concentrations of various pigments within leaves contribute to the diverse range of leaf colors observed [16]. In this study, the yellow leaf mutant exhibited a significant reduction in chlorophyll content compared to the wild type (WT), accompanied by a marked increase in total flavonoid content, while carotenoid levels remained largely unchanged. The ratios of chlorophyll a to chlorophyll b (Chl a/Chl b) and carotenoids to chlorophyll (Car/Chl) were substantially elevated in the mutant relative to the WT. These findings are consistent with observations from studies on other chlorophyll-deficient mutants [13,14]. It can be inferred that the reduction in chlorophyll content, coupled with the increase in flavonoid pigments, constitutes the physiological basis for the yellow leaf (YL) phenotype.

The biosynthesis of chlorophyll is a complex process, involving over a dozen enzymatic reactions to produce chlorophyll a and chlorophyll b [17]. Disruptions at any stage of this pathway can lead to decreased chlorophyll synthesis, ultimately altering leaf coloration [18].

In the current investigation, it was observed that the enzymes associated with chlorophyll biosynthesis and metabolism exhibited alterations at the mRNA level. Glutamyl-tRNA reductase, encoded by the *HEMA* gene, catalyzes the initial step of chlorophyll synthesis and serves as the rate-limiting enzyme in this biosynthetic pathway [19]. Alterations in the expression of the *HEMA* gene can potentially impact chlorophyll synthesis [20]. Consistent with these findings, our study demonstrated that the downregulation of *HEMA* expression inhibited chlorophyll synthesis, resulting in a reduction of chlorophyll content in mutant leaves. Additionally, genes encoding enzymes such as *CHL* [21], *POR* [22] and *DVR* [23] are crucial for chlorophyll biosynthesis, and mutations in these genes lead to chlorophyll deficiency, manifesting as a loss of green pigmentation in plants. The expression of *HEMA*, *HEME*, *PPO*, *CHLI*, *POR*, and *DVR* was downregulated in YL, resulting in the inhibition of chlorophyll synthesis. However, the expression of *GSA*, *HEMD*, and *CAO* was upregulated, suggesting that chlorophyll synthesis was not entirely obstructed, allowing for some chlorophyll accumulation and rendering the YL leaves light green.

Additionally, we observed a significant upregulation of *NYC1* expression in YL. *NYC1* is implicated in the initial step of chlorophyll degradation, specifically catalyzing the degradation of chlorophyll b [24]. The high expression of *NYC1* promoted the degradation of chlorophyll b to chlorophyll a, which may explain the higher ratio of Chl a/Chl b in YL. *SGR*, which plays a crucial role in plant senescence [25], has been shown to promote chlorophyll degradation in *Magnolia sinostellata* leaves under shaded conditions [26]. The expression of *SGR* was notably upregulated in YL, potentially accelerating chlorophyll degradation in the leaves. In addition, the expression of *PAO* and *PPH* was similarly upregulated in YL, and it can be hypothesized that the chlorophyll degradation pathway was remarkably activated. These data suggest that the reduction in chlorophyll content in YL is attributable not only to decreased chlorophyll synthesis but also to increased chlorophyll degradation.

Flavonoid compounds, a class of pigments prevalent in plants, play a significant role in regulating leaf coloration [27]. In the albino tea cultivar “Yu-Jin-Xiang”, several genes within the flavonoid biosynthetic pathway exhibited upregulation, leading to an increased concentration of various flavonoids [28]. This upregulation of the flavonoid synthesis pathway has also been observed in the yellow leaf mutants of *Ulmus pumila* [29] and *Ginkgo biloba* [30]. Consistent with these findings, the current study demonstrated a substantial upregulation of flavonoid biosynthesis in the mutant, characterized by enhanced expression of several upstream flavonoid pathway genes, including *PAL*, *CHS*, and *CHI*. This upregulation resulted in an elevated total flavonoid content in the YL leaves, potentially contributing to their yellowish-green phenotype.

### 3.2. Aberrant Structure of Chloroplasts Affects Chlorophyll Synthesis

Chloroplasts serve as the primary sites for the capture of light energy in plants, facilitated by chlorophyll during the process of photosynthesis [31]. Typically, chloroplasts possess a complex membrane system characterized by numerous grana, which consist of stacks of thylakoids [32]. It has been observed that chlorophyll-deficient mutants frequently exhibit abnormal chloroplast structures [33,34]. Ultrastructural analysis of chloroplasts revealed that in the YL mutant, the chloroplasts’ double membrane was compromised, and the thylakoids were irregularly arranged. Compared to wild-type (WT) plants, YL exhibited a reduced number and size of chloroplasts. These structural alterations likely diminish the photosynthetic capacity of YL, as evidenced by a reduction in starch granules within YL chloroplasts. Photosynthesis in higher plants is a synergistic process involving both light-dependent and carbon assimilation reactions, and disruptions in chloroplast structure can impede this process [35]. Photosystem Ⅰ, photosystem Ⅱ, cytochrome b6/f complex and ATP synthase are present in the thylakoid membrane [36]. The vast majority of the photosynthetic pigments bind to the thylakoid membrane proteins to form the light-harvesting complex (LHC) [37]. The LHC captures light energy and passes it on to the PS Ⅰ and PS Ⅱ reaction center complexes, which convert light energy into active chemical energy [38]. Abnormalities in the genes that regulate the photosystem complexes lead to impaired function of the complexes and inhibition of photosynthesis [39]. In yellow-leaved *Lagerstroemia indica*, the expression of genes involved in photosynthesis, such as *PsbB*, *PsbO*, *PsbP* and *PsbQ*, was down-regulated, protein complexes were disassembled, and photosynthetic pigments were degraded along with the disassembly of the protein complexes [40]. Genes in PS Ⅰ, PS Ⅱ and antenna proteins were significantly downregulated in YL, including genes such as *PsaD*, *PsaE*, *PsaF*, *PsbP*, *PsbR*, *PsbQ*, *Lhca* and *Lhcb*. In summary, the chloroplast structure of YL was damaged, and the photosynthesis system may have been disrupted, allowing degradation of chlorophyll aggregated in the light-trapping complex and yellowing of the leaves.

### 3.3. Signaling Pathway Abnormalities

Chloroplasts are semi-autonomous organelles that necessitate a retrograde signaling pathway to facilitate coordinated communication with the nucleus [41]. The Mitogen Activated Protein Kinase (MAPK) signaling pathway plays a pivotal role in regulating various biological processes, including stress responses, hormone signaling, and developmental processes [42]. Research has demonstrated that the MAPK cascade is integral to the signaling interactions between chloroplasts and the nucleus [43]. In rice, the *OsCSL1* gene encodes a MAPK3 protein that is crucial for chloroplast development, potentially regulating the expression of multiple genes associated with chloroplast synthesis. This regulation may lead to impaired chloroplast development and affect leaf greening in rice [44]. In the present study, transcriptome analysis revealed that differentially expressed genes were significantly enriched in the plant MAPK signaling pathway. It is plausible that a similar MAPK-mediated transcriptional regulation occurred in YL, leading to the suppression of chloroplast-related gene expression, diminished chlorophyll synthesis, leading to the yellow–green coloration of the leaves.

During the process of leaf senescence, plants modulate chlorophyll degradation via the ethylene signaling pathway. The transcriptional regulatory factor ORE1 induces the expression of the key ethylene biosynthesis gene *ACS2* during senescence, thereby activating the ethylene-mediated chlorophyll degradation network [45]. Ethylene Insensitive 3 (EIN3), a principal positive regulator of ethylene signaling, facilitates chlorophyll degradation by directly binding to the promoters of *NYE1*, *NYC1* and *PAO*, thus promoting their expression. Additionally, EIN3 can directly target ORE1 to enhance the expression of genes associated with chlorophyll degradation [46]. Many studies have shown that the MKK9–MPK3/MPK6 cascade is involved in the regulation of ethylene biosynthesis and that MAPKs regulate ethylene biosynthesis by controlling the transcription of *ACS* [47,48]. In this study, transcriptome analysis revealed that differentially expressed genes were significantly enriched in plant hormone signalingtransduction. Notably, the expression of the *EIN3* gene was upregulated in YL, indicating that the ethylene-mediated chlorophyll degradation network might be activated, leading to accelerated chlorophyll degradation and leaf chlorosis.

The MAPK cascade reaction plays an important role in plant hormone signaling, and the signaling of various hormones directly or indirectly affects chlorophyll synthesis and degradation [42,49]. In YL, DEGs were conspicuously enriched in the MAPK signaling pathway and plant hormone signaling transduction, and the synergistic interaction between them may reduce chlorophyll synthesis and promote chlorophyll degradation. This imbalance between the chlorophyll synthesis and degradation processes ultimately results in leaf yellowing.

## 4. Materials and Methods

### 4.1. Plant Material

The experimental materials comprised a yellow–green leaf mutant of *Magnolia sinostellata* (YL) and the wild type *Magnolia sinostellata* (WT). YL is a natural mutant found in the breeding process, and its leaf color trait remained stable after grafting and propagation. The YL specimens used in this experiment were grafted and transplanted into the Zhejiang Academy of Forestry in Hangzhou, China. All specimens were cultivated under uniform environmental conditions at the Zhejiang Academy of Forestry in Hangzhou, China, with regular watering and fertilization. In July, when the leaves were mature but not old, the 3rd to 5th mature leaves were harvested from the stems as experimental material. Following collection, the samples were promptly frozen in liquid nitrogen and subsequently stored at −80 °C to facilitate RNA extraction.

### 4.2. Pigment Content

#### 4.2.1. Chlorophylls and Carotenoids

In July, fresh leaves from wild-type (WT) and YL plants were collected in triplicate for the determination of pigment content following the method outlined by Wellburn and Lichtenthaler [50]. Chlorophyll (Chl) and carotenoids (Car) were extracted using a solvent mixture of anhydrous ethanol, acetone, and distilled water in a ratio of 4.5:4.5:1, over a 24 h period in darkness. The pigment concentrations were then quantified based on absorbance readings at 470 nm, 646 nm, and 663 nm using a Vis–UV spectrophotometer (Metash UV-6100, Shanghai, China). The concentrations of chlorophyll a (Chl a, mg/g), chlorophyll b (Chl b, mg/g), and carotenoids (Car, mg/g) were calculated as follows:

C_Chl a_ (mg/L) = 12.21 A663 − 2.59 A646

C_Chl b_ (mg/L) = 20.88 A646 − 4.67A663

C_Car_ (mg/L) = (1000 × 4.37 A470 – 3.27 × C_Chl a_ – 104 × C_Chl b_)/229

Chl a (mg/g) = C_Chl a_ (mg/L) × V (L)/W_fresh_ (g)

Chl b (mg/g) = C_Chl b_ (mg/L) × V (L)/W_fresh_ (g)

Car (mg/g) = C_car_ (mg/L) × V (L)/W_fresh_ (g)

Chl (mg/g) = Chl a(mg/g) + Chl b (mg/g)

#### 4.2.2. Chlorophyll Precursor

The content of 5-aminolevulinic acid (ALA) was determined using the methods described by Roger [51], while porphobilinogen (PBG) and uroporphyrinogen III (Urogen III) were quantified as described by Bogorad [52]. Protoporphyrin IX (Proto IX), Mg-protoporphyrin IX (Mg-Proto IX), and protochlorophyllide (Pchlide) were extracted following the protocol of Yang [53].

#### 4.2.3. Total Flavonoids

The content of total flavonoids was assessed using the sodium nitrite–aluminum nitrate colorimetric method [54], with rutin serving as the standard. A calibration curve was established (Y = 18.036X + 0.0098, r^2^ = 0.9999) using rutin as the reference standard. The results were quantified as milligrams of rutin equivalent per gram of dry weight (mg/g DW) to determine the flavonoid content.

### 4.3. Ultrastructure Microscopy

Utilizing the double fixation method, samples were initially fixed with 2.5% glutaraldehyde followed by 1% osmium tetroxide (O_s_O_4_). Dehydration was achieved using ethanol solutions of increasing concentrations (30%, 50%, 70% and 80%). Post-osmotic treatment, the samples were embedded, sectioned using a ultramicrotome (LEICA, EM UC7 Wiesbaden, HES, Germany) and stained with uranyl acetate and alkaline lead citrate for 5 to 10 min, respectively. Observations were conducted using a Transmission Electron Microscope (Hitachi, H-7650, Tokyo, Japan). This experiment was carried out by the Bio-Ultrastructure Analysis Laboratory at the Analysis Center of Agrobiology and Environmental Sciences, Zhejiang University, Hangzhou, China.

### 4.4. RNA Extraction, Library Construction, and RNA–Seq

Three biological replicates of both wild type (WT) and YL were prepared for transcriptome sequencing. Sequencing libraries were constructed using the NEBNext^®^ Ultra™ RNA Library Prep Kit for Illumina^®^ (NEB, Beverly, MA, USA) in accordance with the manufacturer’s instructions, and index codes were incorporated to assign sequences to each sample. The libraries were sequenced on an Illumina NovaSeq 6000 platform by the Biomarker Technology Company (Beijing, China).

### 4.5. Gene Annotation and Differentially Expressed Genes (DEGs) Analysis

Gene functions were annotated utilizing the Nr, Swiss-Prot, Kyoto Encyclopedia of Genes and Genomes (KEGG), Eukaryotic Orthologous Groups (KOG), Clusters of Orthologous Groups (COG), and Gene Ontology (GO) databases. The expression level of each gene was quantified using Fragments Per Kilobase of transcript per Million mapped reads (FPKM). Differentially expressed genes (DEGs) between each pairwise sample comparison were identified using a threshold of fold change ≥ 2 and a false discovery rate (FDR) < 0.01, based on statistical analyses conducted with EBSeq.

### 4.6. Quantitative qRT–PCR (qPCR)

Total RNA (in micrograms) extracted from wild-type (WT) and YL samples was utilized for complementary DNA (cDNA) synthesis employing the Fastking RT Kit with gDNase (TIANGEN, Beijing, China) and oligo (dT) primers. Primer sets were designed using the Primer Premiere 5 software (Premiere Biosoft, Palo Alto, CA, USA). Quantitative PCR (qPCR) was conducted on the QIAquant 96 2 plex system (QIAGEN, Hilden, Germany) using the FastReal qPCR PreMix (SYBR Green) (TIANGEN, Beijing, China). The primers for the internal reference gene were adopted from Qianying Wang [55]. Relative gene expression levels were determined using the 2^−ΔΔCt^ method, where ΔCt represents the difference between the cycle threshold (Ct) values of the target and reference genes.

### 4.7. Statistical Analysis

Data were analyzed using SPSS version 26 statistical software (International Business Machines, Armonk, NY, USA). Significance was determined by one-way analysis of variance (ANOVA), with a *p*-value less than 0.05 considered statistically significant. Linear regression analysis was performed using Origin software version 2024 (OriginLab, Northampton, MA, USA) to obtain correlation coefficients (R^2^) and equations.

## 5. Conclusions

In this study, we investigated the underlying causes of the yellow leaf phenotype in *Magnolia sinostellata* by employing physiological parameter measurements, cytological observations, and transcriptomic analysis. Our findings indicated a significant reduction in chlorophyll content in the yellow leaves (YL), accompanied by increased Chl a/Chl b and Car/Chl ratios, as well as a substantial increase in total flavonoid content. These results suggest that alterations in pigment content and ratios are key physiological contributors to the yellow leaf phenotype. Cytological observations further revealed defective chloroplast structures in YL, indicating that impaired chloroplast development may also contribute to the yellowing of leaves. Transcriptomic analysis identified differentially expressed genes enriched in pathways related to chlorophyll metabolism, photosynthesis, flavonoid synthesis, plant hormone signal transduction, and the MAPK signaling pathway. These findings suggest that the yellow leaf phenotype in YL may be attributed to changes in gene expression within these critical pathways.

These findings elucidate the potential mechanisms underlying leaf color formation in the yellow leaf mutant of *Magnolia sinostellata*. Consequently, this research enhances our comprehension of the processes responsible for the yellow leaf phenotype in *Magnolia sinostellata*. Furthermore, it establishes a foundational basis for subsequent investigations into the mechanisms of leaf coloration in other woody plant species.

## Data Availability

Data are contained within the article.
